# The first BILGENSA Research Network workshop in Zambia: identifying research priorities, challenges and needs in genital bilharzia in Southern Africa

**DOI:** 10.12688/wellcomeopenres.22429.1

**Published:** 2024-07-10

**Authors:** Rhoda Ndubani, Olimpia Lamberti, Anna Kildemoes, Pytsje Hoekstra, Jennifer Fitzpatrick, Helen Kelly, Bellington Vwalika, Bodo Randrianasolo, Amy Sturt, Seke Kayuni, Augustine Choko, Nkatya Kasese, Eyrun Kjetland, Takalani Nemungadi, Sibone Mocumbi, Anna Samson, Elizabeth Ntapara, Anifrid Thomson, Elizabeth Danstan, Chido Dziya Chikwari, Kevin Martin, Ibrahim Rabiu, Gifty Terkie, David Chaima, Manuel Kasoka, Karoline Joeker, Louise Thomsen Schmidt Arenholt, Peter Leutscher, Russel Stothard, Oliva Rabozakandria, Anouk Gouvras, Tendai Munthali, Grace Hameja, Paul Kanfwa, Halwindi Hikabasa, Helen Ayles, Kwame Shanaube, Amaya L. Bustinduy

**Affiliations:** 1 Zambart School of Medicine, Lusaka, Zambia; 2 Department of Clinical Research, London School of Hygiene and Tropical Medicine (LSHTM), London, UK; 3 Section for Parasitology and Aquatic Pathobiology, Faculty of Health and Medical Sciences, University of Copenhagen, Copenhagen, Denmark; 4 Department of Parasitology, Leiden University Medical Center, Leiden, The Netherlands; 5 Department of gynaecology, University of Zambia, Lusaka, Lusaka Province, Zambia; 6 Association K'OLO VANOVA, Antananarivo, Madagascar; 7 Infectious Diseases Section, Veterans Affairs Healthcare System, Palo Alto, USA; 8 8. Division of Infectious Diseases and Geographic Medicine, Stanford University, Stanford, California, USA; 9 Department of Tropical Disease Biology, Liverpool School of Tropical Medicine, Liverpool, UK; 10 Discipline of Public Health Medicine, Nelson R Mandela School of Medicine, College of Health Sciences,, University of KwaZulu-Natal, Durban, KwaZulu-Natal, South Africa; 11 Department of Infectious Diseases, Norwegian Centre for Imported and Tropical Diseases, Oslo, Norway; 12 Manhiça Health Research Centre (CISM), Maputo Central Hospital, Maputo, Mozambique; 13 Department of Behavioral Sciences, School of Public Health, Catholic University of Health and Allied Sciences, Mwanza, Tanzania; 14 Mbeya Medical Research Centre (MMRC), National Institute of Medical Research, Mwanza, Tanzania; 15 Biomedical Research and Training Institute, Harare, Harare Province, Zimbabwe; 16 The Centre for Sexual Health and HIV/AIDS Research Zimbabwe, Harare, Zimbabwe; 17 Department of Global Health and Infection, Brighton and Sussex Medical School, Brighton, UK; 18 Department of Community Medicine, Gombe State University, Gombe, Gombe, Nigeria; 19 Department of Pathology, School of Medicine and Oral Health, Kamuzu University of Health Sciences, Blantyre, Malawi; 20 Department of Clinical Medicine, Aalborg University, Aalborg, Denmark; 21 Centre for Clinical Research, North Denmark Regional Hospital, Hjoerring, Denmark; 22 Department of Obstetrics and Gynecology, North Denmark Regional Hospital, Hjoerring, Denmark; 23 Global Schistosomiasis Alliance, London, UK; 24 School of Public Health, University of Zambia, Lusaka, Lusaka Province, Zambia; 25 Department of Public Health, Ministry of Health, Lusaka, Zambia; 26 Department of Neglected Tropical Diseases, Ministry of Health, Lusaka, Zambia

**Keywords:** Female genital schistosomiasis, FGS, male genital schistosomiasis, MGS, Schistosoma haematobium, research, needs, priorities, Southern Africa

## Abstract

Female genital schistosomiasis (FGS) and male genital schistosomiasis (MGS) are gender-specific manifestations of urogenital schistosomiasis. Morbidity is a consequence of prolonged inflammation in the human genital tract caused by the entrapped eggs of the waterborne parasite, *Schistosoma (S.) haematobium.* Both diseases affect the sexual and reproductive health (SRH) of millions of people globally, especially in sub-Sahara Africa (SSA). Awareness and knowledge of these diseases is largely absent among affected communities and healthcare workers in endemic countries. Accurate burden of FGS and MGS disease estimates, single and combined, are absent, mostly due to the absence of standardized methods for individual or population-based screening and diagnosis. In addition, there are disparities in country-specific FGS and MGS knowledge, research and implementation approaches, and diagnosis and treatment. There are currently no WHO guidelines to inform practice. The BILGENSA (Genital Bilharzia in Southern Africa) Research Network aimed to create a collaborative multidisciplinary network to advance clinical research of FGS and MGS across Southern African endemic countries. The workshop was held in Lusaka, Zambia over two days in November 2022. Over 150 researchers and stakeholders from different schistosomiasis endemic settings attended. Attendees identified challenges and research priorities around FGS and MGS from their respective countries. Key research themes identified across settings included: 1) To increase the knowledge about the local burden of FGS and MGS; 2) To raise awareness among local communities and healthcare workers; 3) To develop effective and scalable guidelines for disease diagnosis and management; 4) To understand the effect of treatment interventions on disease progression, and 5) To integrate FGS and MGS within other existing sexual and reproductive health (SRH) services. In its first meeting, the BILGENSA Network set forth a common research agenda across *S. haematobium endemic* countries for the control of FGS and MGS.

## Introduction

Female and male genital schistosomiasis are gender-specific chronic manifestations of urogenital schistosomiasis, a waterborne parasitic disease caused by the blood fluke *Schistosoma (S.) haematobium* ^1, 2^. Globally, female genital schistosomiasis (FGS) affects an estimated 20–56 million girls and women, mostly in sub-Saharan Africa (SSA) ^2^. Male genital schistosomiasis (MGS), is ^1, 3– 5^ estimated to affect between 1% and 20% of males at risk ^1^. Prevalence of FGS and MGS, is extrapolated from a small number of studies and underestimate the true burden of disease ^1, 2^. To date, only approximately 15,000 girls and women in endemic settings have been assessed for FGS ^1, 3^. MGS is also understudied and only six African countries (Madagascar, Nigeria, Egypt, Zimbabwe, Zambia, and Ghana) have conducted MGS studies.

The FGS and MGS epidemiology is closely related to the distribution and transmission dynamics of the parasite *S. haematobium* ^6, 7^ *.* Individuals are exposed to the parasite through skin-contact with larvae (cercariae) in contaminated freshwater sources ^6, 7^. Inside the human host, parasites mature into adults and reside in the blood vessels where they produce eggs ^6, 7^. While some eggs are excreted in urine, others become lodged in the urogenital and genital organs, inducing granulomatous inflammation, and pathological changes ^2, 8, 9^. This can result in adverse reproductive health outcomes, organ dysfunction, and reproductive morbidity ^1^. Importantly, in cross-sectional studies, there was strong evidence that FGS was associated with prevalent HIV and high-risk (HR-) human papillomavirus (HPV), the primary etiological agent of cervical cancer ^2, 7, 10, 11^. No epidemiological study has evaluated the association between MGS and HIV. *S. haematobium* induced inflammation in the male genital tract is hypothesized to increase HIV-1 viral load shedding in semen and contribute to HIV-1 transmission ^12^. Work from Madagascar showed *S. haematobium* egg excretion in semen is associated with leukocytospermia and elevated inflammatory cytokines ^13^. Further work in participants with MGS showed some evidence of a decline in semen viral load after praziquantel treatment (p = 0.08) ^14^. These findings may lend biological plausibility to an association of MGS with HIV transmission, but further research is needed. Despite these significant health impacts, awareness of FGS and MGS is largely absent in *S. haematobium-*endemic settings.

Clinical manifestations of FGS and MGS are non-specific and often overlap with those of other sexual and reproductive health (SRH) conditions ^2, 15^. Girls and women with FGS report symptoms including abdominal pain, vaginal bleeding, and genital itching ^2^. These are often attributed to other sexually transmitted infections (STIs) by both healthcare workers and the patients, leading to overlooking FGS and unnecessary treatment of STIs ^2^. Symptoms of MGS include changes in sperm consistency, presence of blood in semen, coital and ejaculatory pain, abnormal ejaculates, and erection discomfort or dysfunction ^1^. Compared to FGS, the level of morbidity associated with MGS in endemic areas remains largely understudied, with most of the evidence coming from individual case reports and post-mortem studies ^1^. The stigma and misconception associated with these symptoms further contribute to the challenges of underreporting and incorrect diagnosis of MGS in endemic areas ^1^.

There are no standardized methods for individual or population-based screening and diagnosis of FGS and MGS, resulting in substantial underreporting of their prevalence and associated morbidity ^2, 15, 16^. Conventional diagnosis of FGS involves the use of a colposcope to visually identify FGS associated lesions ^6^. This is costly and requires high-level specialized training and advanced clinical infrastructure, which are often unavailable in *S. haematobium* endemic settings ^6^. In addition, visual diagnosis of FGS may lack specificity, as the FGS mucosal changes visually observed with colposcopy have been associated with malignancy and STIs ^11, 17^. Microscopy of semen samples is currently the most accurate diagnostic method available for MGS ^1^. However, its acceptance and availability in endemic communities is hindered by local beliefs and perceptions, posing significant challenges to its widespread implementation ^1^. More recent research studies in SSA countries have validate closer-to the-user strategies for FGS screening and diagnosis ^15^. Community-based screening and diagnostic strategies at the point-of-care could offer a promising opportunity for surveillance at scale ^2, 15, 18^.

FGS and MGS treatment and control relies on schistosomiasis public health guidelines, which promote mass drug administration (MDA) of praziquantel, as preventive chemotherapy ^19^. Although praziquantel effectively reduces urinary egg excretion, its efficacy in treating genital schistosomiasis remains uncertain due to lack of robust clinical evidence ^2^. WHO recommends the rollout of MDA treatment strategies based on schistosomiasis population-based prevalence, using a 10% threshold to determine the targeted age groups for treatment ^19^. Yet, this approach is likely to overlook individuals affected by FGS and MGS who are not included in the treatment programs, contributing to the ineffective control of FGS and MGS in endemic regions.

In November 2022, the first BILGENSA (Genital Bilharzia in Southern Africa) workshop, took place in Lusaka, Zambia. The aim of the workshop was to advance the field at country-level and as part of a wider strategy for the control of schistosomiasis worldwide. Researchers from various *S. haematobium* endemic countries gathered to discuss the research gaps and needs for FGS and MGS research. This paper reports the key research needs and priorities that emerged during the workshop.

## The BILGENSA Research Network

The BILGENSA Research Network was a multi-country workshop held on the 9 ^th^ and 10 ^th^ of November 2022 in Lusaka, Zambia. The workshop aimed to establish a collaborative multidisciplinary network to share expertise and advance clinical research on FGS and MGS across *S. haematobium* endemic countries. The workshop was conducted over two days and used a hybrid approach with delegates and discussion both in-person and virtually. Over 150 researchers specializing in FGS, MGS, HIV, STIs and cervical cancer (CC) from around the globe attended the workshop. Attendees with a specialty in schistosomiasis research represented diverse research areas including disease epidemiology, diagnostics, parasitology, program implementation, and qualitative research on disease awareness. Delegates from *S. haematobium* endemic countries, including Zambia, Zimbabwe, Tanzania, Nigeria, South Africa, Madagascar, Ghana, Malawi, and Mozambique attended the workshop in Lusaka, Zambia.

**Figure 1.**
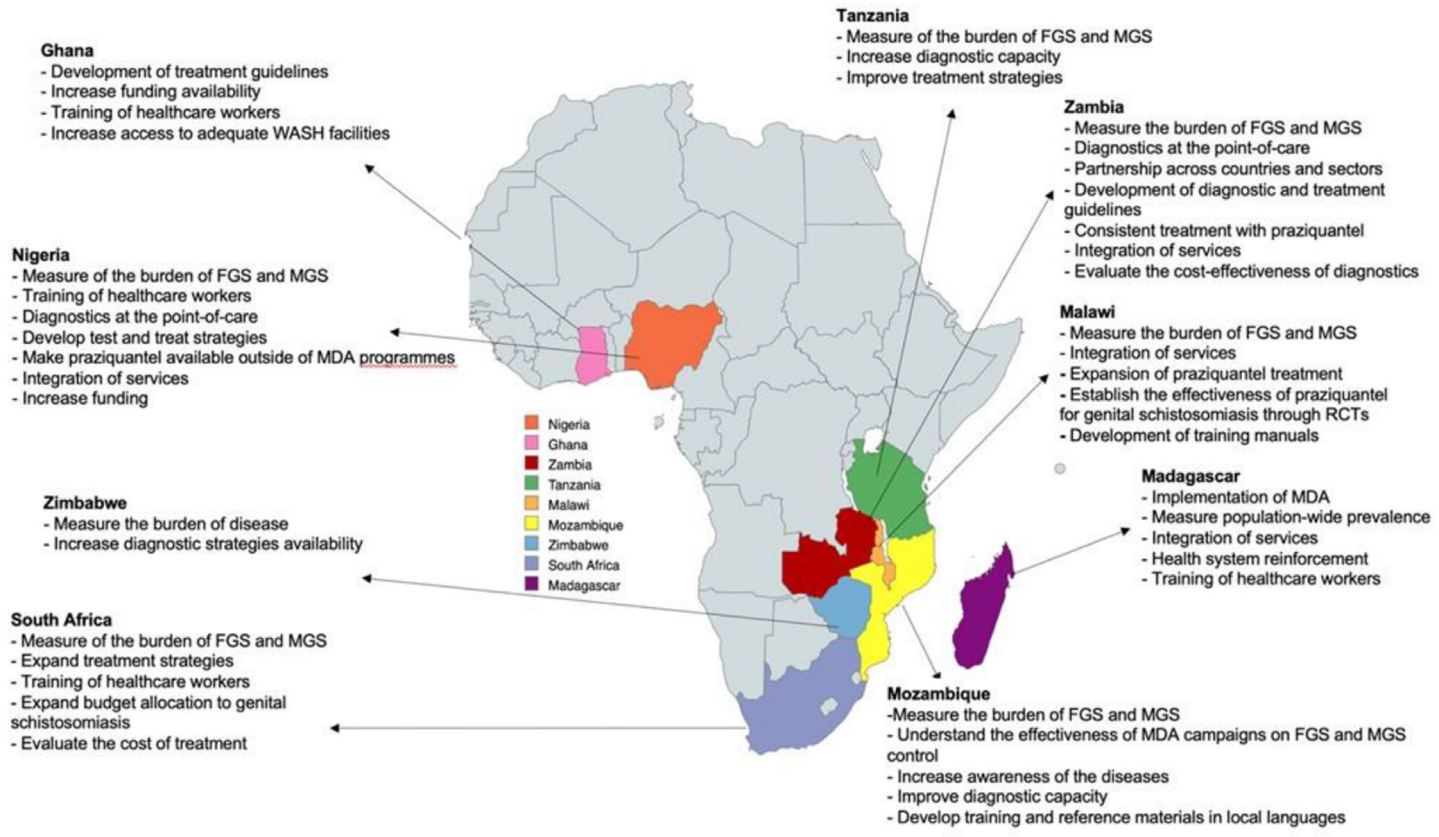
Map showing the countries represented during the BILGENSA Research Network and their research needs and priorities to advance research on female genital schistosomiasis (FGS) and male genital schistosomiasis (MGS).

The event was hosted by Zambart ( https://www.zambart.org.zm), a Zambian research institution with extensive experience in HIV, TB, and SRH research. Sessions were held in English and translated in French for online participants. The workshop’s aim and objectives are presented in *Extended data*, Figure 1 ^20^. The workshop was conducted over two days ( *Extended data,* Text 1). Day one comprised of didactic sessions in which participants presented in-country FGS and MGS research, as well as sharing their country’s perceived research priorities, challenges, and needs. The afternoon sessions were interactive with participants grouped in their respective countries to discuss country-specific research priorities ( *Extended data,* Text 1). At the conclusion of Day One, outcomes of the interactive session were shared with the wider group. During Day Two, participants discussed the diagnostic opportunities and challenges for genital schistosomiasis and integration within SRH services. Specific interventions suggested included integrating FGS within existing cervical cancer, and FGS/MGS within HIV care screening programs ( *Extended data,* Figure 1). We identified common themes that emerged during the workshop.

## Research needs and priorities identified in the workshop

During the BILGENSA workshop, endemic countries reported being at different stages in advancing FGS and MGS research. Despite these differences, common themes identified across countries include lack of expertise in identifying and correctly diagnosing FGS/MGS, and lack of disease awareness. The scarcity of available diagnostics and specialized equipment and health facilities infrastructure are notable hurdles. Another limitation is the limited number of trained clinicians available with specific expertise in FGS screening and identification. All country delegates reported limited availability of routine treatment with praziquantel, a drug not commonly found in clinics in SSA ^21^. Further common issues include lack of financial resources from the ministry of health (MoH) to support community education and mobilization. There is a clear need for *enhanced collaboration* across countries and sectors.

Following the identification of the above challenges, the research priorities, and strategies to address them set forth by country delegates, as follows ( Figure 1/ Table1).

**Table 1.** Summary of the recommendations for future research discussed during the BILGENSA Research Network to address the female genital schistosomiasis (FGS) and male genital schistosomiasis (MGS) research needs and priorities in endemic settings.

Research need	Recommendations for future research	
	FGS	MGS
**Raising awareness and ** **knowledge**	Provide further training to healthcare workers and patients on etiology, transmission, symptoms, screening, and management	Develop and disseminate educational and training material
**Improving diagnostic ** **capabilities**	Validate and implement decentralized and field- deployable screening and diagnostic strategies for community-based surveillance at scale. Evaluate the scalability and cost-effectiveness of different screening and diagnostic strategies	Develop and validate accessible, scalable, and low-cost molecular test.
**Developing standardized** ** treatment guidelines**	Conduct randomized control trials (RCTs) to evaluate the effectiveness of praziquantel for FGS and MGS Develop and implement FGS/MGS specific treatment guidelines.	
**Integration of SRH ** **screening strategies**	Evaluate the integration of FGS within the wider schistosomiasis and SRH control guidelines.	Identify potential opportunities for integration of MGS into ongoing HIV interventions
**Surveillance and ** **monitoring**	Develop routine and integrated surveillance of FGS Increase collaborative efforts and financial resources available to implement and expand FGS and MGS control strategies.	

### Raising knowledge and awareness of FGS and MGS

During the workshop, delegates highlighted the urgent need to increase awareness and education on FGS and MGS in endemic settings through community engagement and sensitization programs. Community-wide campaigns should be initiated to disseminate information on the diseases’ etiology, modes of transmission, symptoms, and prevention strategies. These campaigns can leverage existing community structures and engagement programs, to ensure all members of the community are included. It is imperative to provide additional training to healthcare workers in *S. haematobium* endemic regions on the acquisition, screening, and treatment of FGS and MGS. This training could be integrated into ongoing educational programs for Neglected Tropical Diseases (NTDs) and SRH ^3, 22^. The development of training manuals, standard operating procedures (SOPs) and standardized guidelines for the diagnosis and treatment of FGS and MGS are necessary to ensure effective disease control. An FGS training document for the community and healthcare workers, as well as a manual about raising awareness on FGS using drama, have recently been developed as a first step towards increasing education about and information on the disease, respectively ^23– 25^. In contrast, the lack of knowledge and awareness of MGS has not yet been adequately addressed.

Recent qualitative research in Cameroon, Tanzania, and Ghana highlighted a significant gap in knowledge and awareness on FGS among communities and healthcare workers ^26– 28^. Girls and women have reported limited knowledge on the mode of transmission, symptoms, and potential risk factors associated with FGS ^26– 28^. Simultaneously, healthcare workers often incorrectly diagnosed FGS, confusing its symptoms with those of other STIs ^26– 28^. This results in the unnecessary STI treatment and contributes to the stigma faced by affected individuals seeking care in public health facilities ^2^. Immediate action is necessary to implement further programs aimed at raising awareness and disseminating information and education on FGS among affected populations and healthcare workers, to effectively control and manage schistosomiasis. Further work is needed to develop educational material for MGS.

### Improving diagnostic capabilities in-country

Many countries reported limited diagnostic capacity for FGS and MGS with some (such as Nigeria, Tanzania, and Malawi) relying solely on *S. haematobium* antigen, antibody, and pathogen-based diagnosis. Although these methods serve as a useful proxy, they have reduced sensitivity for FGS and MGS diagnosis as they do not confirm genital involvement ^1, 2^. An additional common challenge identified across countries was the limited number of clinicians trained in colposcopy to identify FGS specific lesions, leading to a bottleneck in diagnosis.

Given the limited resources available in *S. haematobium* endemic countries, the BILGENSA Research Network highlighted the importance of decentralizing diagnostic methods for FGS. This includes bringing screening closer to the user for community-based surveillance at scale ^2^. *Hand-held colposcopy* has been proposed as a more scalable diagnostic strategy compared to traditional colposcopy ^2, 17, 29^. Hand-held colposcopes can be operated by primary healthcare workers (midwives), are mobile, cheaper and, rechargeable ^2, 17, 29^. Furthermore, novel approaches for analysis of colposcopic images include using artificial intelligence visual reading algorithms to overcome some of the FGS diagnostic barriers including the lack of trained clinicians to identify FGS lesions and the subjectivity of FGS visual diagnosis ^29^. Community-based screening and testing using *home-based self-sampling* and *point-of-care (POC) diagnostics* has also been proposed as a closer-to-the-user strategy for surveillance at scale ^15^. Previous studies have shown the feasibility, acceptability, and cost-effectiveness of home-based genital self-sampling for cervical cancer screening, which has the potential to increase coverage by reaching women less likely to attend screening in clinic. A recent study in Zambia validated the use of genital self-sampling as a feasible, accurate, and feasible method for community-based screening of FGS ^15, 30^. As part of the study they successfully piloted a rapid and portable recombinase polymerase assay (RPA) for FGS diagnosis ^15, 31^. The RPA is a POC molecular assay which has high specificity, meaning detecting *S. haematobium* DNA in genital specimen confirms an FGS diagnosis ^31, 32^. The promising results from these studies emphasize the need for further applications of these screening and diagnostic techniques across different endemic settings.

In contrast to FGS, there is limited research on novel MGS screening and diagnostic methods ^5^. Accessible and low-cost molecular tests are urgently needed to address the diagnostic challenges associated with MGS. For both FGS and MGS, future research should evaluate the cost-effectiveness, scalability, and performance of field-deployable molecular assays designed for point-of-care applications. These efforts are crucial for advancing the implementation of decentralized diagnostic strategies, ultimately contributing to more effective and accessible community-based surveillance of FGS and MGS.

### Developing standardized treatment guidelines

Treatment recommendations for FGS and MGS follow schistosomiasis public health guidelines, which promote mass drug administration (MDA) of praziquantel (typically offered at 40mg/kg), as preventive chemotherapy ^2^. Praziquantel is an effective treatment for urinary schistosomiasis, but the evidence on its effectiveness for treating FGS and MGS remains limited. To date, only a small number of observational studies evaluated the performance of praziquantel for the treatment of FGS, and there have been no studies on its effectiveness for MGS ^2^. In endemic settings, treatment programs are determined by the schistosomiasis prevalence ^19^. The World Health Organization (WHO) recommends annual praziquantel treatment for all individuals aged two years and older in communities where the prevalence of egg-patent *Schistosoma* infection is 10% or greater ^19^. In contrast, for communities with a prevalence below 10%, a test-and-treat strategy is advised ^19^. Despite these guidelines, some countries, such as Mozambique and Tanzania, reported that MDA programs exclusively target school-aged-children (SAC), leaving older individuals and adults with limited access to treatment. Additionally, access to and uptake of praziquantel still remain a challenge ^33^

During the BILGENSA workshop, participants highlighted the need to expand FGS and MGS treatment strategies. There was a call for randomized control trials (RCTs) to evaluate the effectiveness of praziquantel for the treatment of FGS and MGS ^2^. Results from these trials will play a pivotal role in the development of effective treatment protocols and guidelines ^2^. Mapping surveys should also be conducted to assess the impact of MDA programs on the prevalence of FGS and MGS in endemic settings and to facilitate an assessment of MDA coverage in rural areas and among hard-to-reach-populations. These efforts are critical for improving treatment accessibility and effectiveness of control strategies in endemic regions.

### Integration of SRH screening strategies

FGS and MGS surveillance are not yet included in wider schistosomiasis or SRH control strategies ^2, 3^. Existing HIV and cervical cancer programs have been identified as opportunities to integrate FGS surveillance into the SRH agenda ^2, 3, 18^. Healthcare delivery systems already in place for HIV and cervical cancer prevention and control can be used to increase access to FGS screening and treatment services ^2, 3, 18^. An integrated home-based screening and testing package for different SRH conditions, including FGS, could be made available to individuals in *S. haematobium* endemic settings ^2, 15^. This is currently being validated in an ongoing study in Zambia which aims to assess the feasibility and acceptability of an integrated home-based approach for multi-pathogen genital screening, including FGS, HPV, Trichomonas and HIV ^34^. In addition, FGS screening using hand-held colposcopy could be integrated into the existing cervical cancer screening programs ^17, 35^. In contrast, for men, there is still a need to identify potential opportunities for integration of MGS screening and control into ongoing HIV interventions. An integrated approach presents an opportunity to increase screening coverage of genital schistosomiasis while developing comprehensive policy frameworks that addresses the disease burden of FGS, MGS, HIV, and cervical cancer. Ultimately, this will accelerate the attainment of universal health coverage by strengthening different levels of the health system.

### Surveillance and monitoring

FGS has been financially neglected, resulting in limited funding available for advancing research and control of these diseases across endemic settings. The shift from campaign-based programs to routine and integrated surveillance of FGS requires further financial resources and sustainable collaborations between sectors. Across countries, participants of the BILGENSA Research Network highlighted the need to increase collaborative efforts and financial resources available to implement and expand FGS control strategies. SRH programs have been proposed as a plausible platform for integration of FGS surveillance within national health systems and information systems. This still requires improved FGS diagnostics, and better understanding of the spatial distribution of disease.

## Conclusions

FGS and MGS are specific genital tract manifestations of urogenital schistosomiasis. These diseases have been largely neglected and under-researched in *S. haematobium* endemic countries. The BILGENSA Research Network was created to bring together researchers and experts from different Southern African countries working on FGS, MGS, within the wider SRH landscape. Common key priorities included improving awareness and knowledge around genital schistosomiasis, increasing surveillance-at-scale by developing decentralized screening and diagnostic guidelines, and exploring the effectiveness of integration of control strategies within the broader schistosomiasis and SRH agendas. These actions are paramount for the control and elimination of these diseases in affected communities.

## Data Availability

No underlying data are associated with this study. Zenodo: The first BILGENSA Research Network Workshop in Zambia; Identifying Research Priorities, Challenges and Needs in Genital Bilharzia in Southern Africa. https://doi.org/10.5281/zenodo.11930644 ^20^ This project contains the following extended data: Supplementary document_12.06.2024.docx (Figure 1: Schematic figure presenting the aims and objectives of the BILGENSA Research Network, Text 1: Description of the proposed activities conducted during the BILGENSA Research Network, Table 1: Agenda for the BILGENSA Research Network) Data are available under the terms of the Creative Commons Attribution 4.0 International license (CC-BY 4.0).
