# Supplementary material for: The first BILGENSA Research Network workshop in Zambia: identifying research priorities, challenges and needs in genital bilharzia in Southern Africa
Source: Wellcome Open Res. 2025 Apr 11;9:360. Originally published 2024 Jul 10. [Version 2] doi: 10.12688/wellcomeopenres.22429.2 (PMC11336559; doi:10.12688/wellcomeopenres.22429.2)
Supplement: Peer review history

# Reviewer report: Reviewer response for version 2

**DOI:** 10.21956/wellcomeopenres.26419.r122125

**Published:** 2025-04-26

**Authors:** Joseph Osarfo

**Affiliations:** 1 Department of Community Health, University of Health and Allied Sciences, Ho, Ghana

have looked at it and I am content that it represents an improvement over the initial submitted manuscript.

 I am grateful to the authors for addressing my comments.....although I still think the last sentence in the second paragraph of the 'Introduction' should have been referenced.

Does the article adequately reference differing views and opinions?

Yes

Are all factual statements correct, and are statements and arguments made adequately supported by citations?

Partly

Is the Open Letter written in accessible language?

Yes

Where applicable, are recommendations and next steps explained clearly for others to follow?

Yes

Is the rationale for the Open Letter provided in sufficient detail?

Yes

Reviewer Expertise:

NA

I confirm that I have read this submission and believe that I have an appropriate level of expertise to confirm that it is of an acceptable scientific standard.

# Reviewer report: Reviewer response for version 1

**DOI:** 10.21956/wellcomeopenres.24710.r91915

**Published:** 2024-08-21

**Authors:** Leora N. Pillay

**Affiliations:** 1 Frontline AIDS, Cape Town, South Africa

The open letter summarises the BILENGSIA workshop held in 2022 and speaks to the research needs that were identified by countries. It is an interesting summary of what was discussed at this workshop and highlights the research questions and needs identified by countries.

 Introduction:

 The introduction is comprehensive and provides a good summary of MGS and FGS. 

 The intro speaks to how gaining semen samples from men to test for MGS is hindered by cultural beliefs and perceptions, posing challenges. A gap identified in the introduction is that nowhere is it acknowledged the invasiveness of colposcopy and how cultural beliefs can impact the screening and diagnosis of FGS.

 The introduction also lacks important information on FGS as a gendered issue as a result of the disproportionate risk that women and girls face due to the split of household chores. 

 Research needs and priorities identified in the workshop:

 The first need identified was 'Raising knowledge and awareness of FGS and MGS' which included the proposed topics for community awareness and sensitisation. These topics are purely clinical around the disease's etiology, modes of transmission, symptoms and prevention strategies but do not tackle stigma, discrimination around the overlap with STI symptoms and the possibility of gender based violence for women and girls. Whilst this may not have come up in the workshop itself, it should be acknowledged as a gap and must be included in any community awareness of FGS.

 Additionally, integration with NTDs and SRH is mentioned but there seems to be no mention of WASH as part of work on prevention in communities and community awareness which is concerning. These gaps should be discussed somewhere in the open letter. 

 Integration with HIV programmes for both MGS and FGS came up in the workshop, but thinking around how this integration could take place for MGS seems really lacking and something to highlight as needing action.

 Information and health literacy on MGS seems to be a large gap but the open letter doesn't go into detail as to why this is the case and what the proposed actions are.

 Improving diagnostic capabilities in country:

 An important element to note as a gap is the invasive way that FGS that is diagnosed for women and girls. We should be searching for new ways to diagnose FGS without a colposcopy, but also ensure that it is acknowledged and information is given to women about the process etc. to ease any concerns/worries.

 Conclusion

 The conclusions section should sum up the workshop discussions but also acknowledge remaining gaps and possible next steps.

Does the article adequately reference differing views and opinions?

Partly

Are all factual statements correct, and are statements and arguments made adequately supported by citations?

Yes

Is the Open Letter written in accessible language?

Yes

Where applicable, are recommendations and next steps explained clearly for others to follow?

Partly

Is the rationale for the Open Letter provided in sufficient detail?

Yes

Reviewer Expertise:

I lead advocacy for FGS integration.

I confirm that I have read this submission and believe that I have an appropriate level of expertise to confirm that it is of an acceptable scientific standard, however I have significant reservations, as outlined above.

# Reviewer report: Reviewer response for version 1

**DOI:** 10.21956/wellcomeopenres.24710.r91910

**Published:** 2024-08-20

**Authors:** W. Evan Secor

**Affiliations:** 1 Centers for Disease Control and Prevention, Atlanta, Georgia, USA

The formation of the BILGENSA Research Network and the accompanying report on the first workshop are very encouraging to see. The workshop participants have been deeply engaged in female and male genital schistosomiasis and identified important priorities for future work. The report on the workshop reads well although some light copy editing could be in order to correct typographical errors and reduce redundancies that likely resulted from different authors for the different sections. The only thing really missing from the report is "what's next" for the BILGENSA Research Network to keep the momentum up. Are there plans for another workshop in the coming year or perhaps meeting in conjunction with another planned conference? There appears to be an intent to develop harmonized protocols for field-deployable screening and diagnostics strategies; how will these be shared with other researchers and ministries of health? One other small question--how are men defined to be "at risk" of MGS (introductory paragraph)?

Does the article adequately reference differing views and opinions?

Yes

Are all factual statements correct, and are statements and arguments made adequately supported by citations?

Yes

Is the Open Letter written in accessible language?

Yes

Where applicable, are recommendations and next steps explained clearly for others to follow?

Partly

Is the rationale for the Open Letter provided in sufficient detail?

Yes

Reviewer Expertise:

Multiple aspects of laboratory and field research on schistosomiasis.

I confirm that I have read this submission and believe that I have an appropriate level of expertise to confirm that it is of an acceptable scientific standard.

# Reviewer report: Reviewer response for version 1

**DOI:** 10.21956/wellcomeopenres.24710.r91907

**Published:** 2024-08-15

**Authors:** Ogechukwu Benedicta Aribodor

**Affiliations:** 1 Nnamdi Azikiwe University, Awka, Nigeria

General Remarks:

 This manuscript offers significant insights into the research priorities, challenges, and needs related to genital bilharzia (schistosomiasis) in Southern Africa and other *Schistosoma haematobium*-endemic countries.  The first BILGENSA Research Network workshop highlighted critical areas requiring attention and advancement. However, several corrections and additions are needed to enhance clarity and accuracy:

**Full Meaning of BILGENSA:** The acronym BILGENSA is introduced in the title. Providing the full meaning of BILGENSA would guide readers and enhance understanding of the network’s scope and relevance.

**Terminology in the Abstract:** The term 'waterborne' used to describe *Schistosoma haematobium* is incorrect. Please rephrase that the infection is associated with snails infected with *S. haematobium* in aquatic habitats, or use 'freshwater parasite'. This correction should be made consistently throughout the manuscript.

**WHO Guidelines: **The statement regarding the absence of WHO guidelines for female genital schistosomiasis is inaccurate. Refer to the WHO document Female Genital Schistosomiasis: A Pocket Atlas for Clinical Health-Care Professionals. While guidelines for female genital schistosomiasis are available, there are no established guidelines for male genital schistosomiasis (MGS).

**Diagnostic Challenges for MGS: **It is important to discuss the variability in clinical manifestations of MGS. The diverse presentation of symptoms, which can be subtle or overlap with other conditions, complicates the development of standardized diagnostic approaches. Highlighting these challenges provides crucial context for understanding why standardization in MGS diagnosis is lacking.

**Impact of Stigma and Misconceptions:** Include a discussion on how stigma and misconceptions related to the symptoms of MGS and FGS further complicate accurate diagnosis and reporting. These factors contribute to underreporting and incorrect diagnoses in many endemic areas. Additionally, cultural and religious constraints, especially where discussing female genital issues is taboo, exacerbate these challenges. For further information, refer to [Aribodor OB *et al. *(2024 ^1^ ].

**Diagnostic Methods for FGS:** Note that invasive diagnostic methods such as colposcopy are generally used only for sexually active girls and women. Jacobson et al. (2021) suggest employing syndromic diagnosis for virgins. The reliance on invasive techniques can be a significant barrier to diagnosing FGS in those who do not meet the criteria for these procedures.

**Adolescent Communication Preferences:** Address the role of adolescents' preferences in discussing their sexual health. (Aribodor OB *et al. *(2023 ^2^ ). Reluctance to share sensitive information due to privacy concerns, stigma, or lack of trust can impede accurate diagnosis and treatment of FGS and MGS. Improving communication barriers is essential for effective diagnostic practices and care.

**FAST Package Training Manual:** Refer to the training manual developed by the FAST package (https://fastpackage.org/), which provides valuable insights for diagnosing FGS. However, it is noted that this manual does not cover MGS, highlighting the need for additional resources and standardization efforts specifically for MGS.

**Need for Low-Cost Molecular Tests: **Emphasize the urgent need for accessible, low-cost molecular tests for MGS that can function without electricity, given the frequent power supply issues in schistosomiasis-endemic communities. Such tests would greatly improve diagnostic capabilities in resource-limited settings and enhance health outcomes.

 Thank you.

Does the article adequately reference differing views and opinions?

Yes

Are all factual statements correct, and are statements and arguments made adequately supported by citations?

Partly

Is the Open Letter written in accessible language?

Yes

Where applicable, are recommendations and next steps explained clearly for others to follow?

Yes

Is the rationale for the Open Letter provided in sufficient detail?

Yes

Reviewer Expertise:

Parasitology and Public Health Parasitology with a specific focus on schistosomiasis. I have extensive experience in researching the epidemiology, diagnostics, and treatment of schistosomiasis, particularly Female Genital Schistosomiasis (FGS). I am familiar with the challenges faced in endemic regions and have actively contributed to the development of FGS competencies, having been trained as a scholar in this area. My expertise enables me to critically evaluate methodologies and findings related to schistosomiasis research and to provide valuable insights aimed at improving diagnostic and treatment approaches.

I confirm that I have read this submission and believe that I have an appropriate level of expertise to confirm that it is of an acceptable scientific standard, however I have significant reservations, as outlined above.

## References

[1] : Assessing urogenital schistosomiasis and female genital schistosomiasis (FGS) among adolescents in Anaocha, Anambra State, Nigeria: implications for ongoing control efforts. *BMC Public Health* .2024;24(1) : 10.1186/s12889-024-18378-0 952 10.1186/s12889-024-18378-0 38566137 PMC10988871

[2] : Profiling the knowledge of female medical/para-medical students, and expertise of health care professionals on female genital schistosomiasis in Anambra, South Eastern Nigeria. *PLoS Negl Trop Dis* .2023;17(2) : 10.1371/journal.pntd.0011132 e0011132 10.1371/journal.pntd.0011132 36795786 PMC9977039

# Reviewer report: Reviewer response for version 1

**DOI:** 10.21956/wellcomeopenres.24710.r91912

**Published:** 2024-08-09

**Authors:** Joseph Osarfo

**Affiliations:** 1 Department of Community Health, University of Health and Allied Sciences, Ho, Ghana

**General Comments**

 I think this report presents a true reflection of challenges, needs and research priorities in genital schistosomiasis in the participating endemic countries (although I suspect Ghana would benefit also from studies to evaluate the burden of FGS and MGS). It is well written and I recommend it is accepted for indexing after the authors have addressed the few comments below;

**Abstract**

1. “ *Accurate burden of FGS and MGS disease estimates, single and combined, are absent, mostly due to the absence of standardized methods for individual or population-based screening and diagnosis*.”
  I daresay that a bigger problem is whether it is diagnosed at all, especially FGS....before a debate on the methods of diagnosis ensues. I think the sentence preceding the extracted text above supports my point.
2. *“Morbidity is a consequence of prolonged inflammation in the human genital tract caused by the entrapped eggs of the waterborne parasite, Schistosoma (S.) haematobium.”*
  Describing ‘Schisto’ as waterborne poses a problem. ‘Waterborne’ will typically be used to describe an infection obtained from drinking water contaminated with human faeces or urine. Schistosomiasis is described as ‘Water-based’ as the parasite lives at some point in an intermediate host that lives in water.

**Introduction**

1. *“......Others become lodged in the urogenital and genital organs,....”*  I would think the term ‘urogenital’ covers both the urinary and genital pathways and repeating ‘genital organs’ is somewhat of a tautology.
2. 2 ^nd^ paragraph, last sentence needs citation(s).
3. “Symptoms of MGS include changes in sperm consistency,.......” Is it sperm consistency or semen consistency??  
4. Are the authors aware of the following education and training document on schisto?
  Schistosomiasis and Female Genital Schistosomiasis (FGS): a booklet for educators [Internet]. TheFAST Package; 2022. Available from: eliminateschisto.org , https://fastpackage.org/wp-content/uploads/2022/04/Teachers_Guide_FAST_Package.pdf

Does the article adequately reference differing views and opinions?

Yes

Are all factual statements correct, and are statements and arguments made adequately supported by citations?

Partly

Is the Open Letter written in accessible language?

Yes

Where applicable, are recommendations and next steps explained clearly for others to follow?

Yes

Is the rationale for the Open Letter provided in sufficient detail?

Yes

Reviewer Expertise:

infectious diseases and epidemiology, health systems strengthening, malaria in pregnancy

I confirm that I have read this submission and believe that I have an appropriate level of expertise to confirm that it is of an acceptable scientific standard, however I have significant reservations, as outlined above.
